# Unique Prokaryotic Consortia in Geochemically Distinct Sediments from Red Sea Atlantis II and Discovery Deep Brine Pools

**DOI:** 10.1371/journal.pone.0042872

**Published:** 2012-08-20

**Authors:** Rania Siam, Ghada A. Mustafa, Hazem Sharaf, Ahmed Moustafa, Adham R. Ramadan, Andre Antunes, Vladimir B. Bajic, Uli Stingl, Nardine G. R. Marsis, Marco J. L. Coolen, Mitchell Sogin, Ari J. S. Ferreira, Hamza El Dorry

**Affiliations:** 1 Biotechnology Graduate Program, American University in Cairo, Cairo, Egypt; 2 Department of Biology, American University in Cairo, Cairo, Egypt; 3 The Science and Technology Research Center, American University in Cairo, Cairo, Egypt; 4 Department of Chemistry, American University in Cairo, Cairo, Egypt; 5 Red Sea Research Center, King Abdullah University of Science and Technology (KAUST), Thuwal, Kingdom of Saudi Arabia; 6 Computational Bioscience Research Center (CBRC), King Abdullah University of Science and Technology (KAUST), Thuwal, Kingdom of Saudi Arabia; 7 Marine Chemistry and Geochemistry Department, Woods Hole Oceanographic Institution, Woods Hole, Massachusetts, United States of America; 8 Josephine Bay Paul Center, Marine Biological Laboratory, Woods Hole, Massachusetts, United States of America; Missouri University of Science and Technology, United States of America

## Abstract

The seafloor is a unique environment, which allows insights into how geochemical processes affect the diversity of biological life. Among its diverse ecosystems are deep-sea brine pools - water bodies characterized by a unique combination of extreme conditions. The ‘polyextremophiles’ that constitute the microbial assemblage of these deep hot brines have not been comprehensively studied. We report a comparative taxonomic analysis of the prokaryotic communities of the sediments directly below the Red Sea brine pools, namely, Atlantis II, Discovery, Chain Deep, and an adjacent brine-influenced site. Analyses of sediment samples and high-throughput pyrosequencing of PCR-amplified environmental 16S ribosomal RNA genes (16S rDNA) revealed that one sulfur (S)-rich Atlantis II and one nitrogen (N)-rich Discovery Deep section contained distinct microbial populations that differed from those found in the other sediment samples examined. *Proteobacteria*, *Actinobacteria*, *Cyanobacteria*, *Deferribacteres*, and Euryarchaeota were the most abundant bacterial and archaeal phyla in both the S- and N-rich sections. Relative abundance-based hierarchical clustering of the 16S rDNA pyrotags assigned to major taxonomic groups allowed us to categorize the archaeal and bacterial communities into three major and distinct groups; group I was unique to the S-rich Atlantis II section (ATII-1), group II was characteristic for the N-rich Discovery sample (DD-1), and group III reflected the composition of the remaining sediments. Many of the groups detected in the S-rich Atlantis II section are likely to play a dominant role in the cycling of methane and sulfur due to their phylogenetic affiliations with bacteria and archaea involved in anaerobic methane oxidation and sulfate reduction.

## Introduction

The Red Sea represents a 450,000-km^2^ inlet of the Indian Ocean located between the African continent and the Arabian Peninsula. Among the most intriguing ecological niches in the Red Sea are its deep-sea brines, which exhibit unique and diverse geochemical conditions [1]. Twenty-five brine pools have been described to date in the Red Sea [2], however, our knowledge of the microbial communities that inhabit these pools, and how these organisms are affected by and contribute to the geochemical properties of their extreme environments, remains sparse.

For example, the hot brine system at Atlantis II Deep (ATII) is the largest and best characterized pool in the Red Sea. Located at a depth of approximately 2200 m near the Central Rift (at around 21°20′ N), it is less than 100 m thick, and covers an area of 60 km^2^ [3]–[5]. The combination of anoxia and high pressure, increased temperature (68°C) and salinity (250 parts per thousand or 7.5 times that of normal seawater [6]) in the brine pool makes it an ideal setting for the study of extremophiles. The Atlantis II Deep is the largest marine sulfide deposit currently known and contains high concentrations of manganese, iron, molybdenum, cadmium, cobalt, copper, nickel, lead and zinc, as well as methane and other hydrocarbons [7]–[8]. The brine is stratified, with the lower convective layer (LCL) exhibiting the highest temperature and salinity, while the three upper convective layers (UCL1, 2 and 3) display step-wise drops in salinity and temperature [4]. Discovery Deep (DD) is likely linked to Atlantis II via subsurface connections [9], [10]. However, the lack of documented long-term temperature variations, suggests the absence of hydrothermal activity at this site [11]. The conditions in the Discovery Deep brine are less harsh and not as well described as Atlantis II, with relatively high concentrations of metals and a milder temperature of 44.8°C [12].

The microbial life found in the hot brine pools in the Red Sea has been little studied owing to technical constraints [reviewed in [12]. Microbial diversity studies based on cultivation-dependent and cultivation–independent approaches have focused mostly on the colder, northern brine pools of the Red Sea. These surveys have revealed an unexpected microbial diversity, including the identification of several new deeply branching phylogenetic lineages, and the isolation and description of several new unusual extremophiles [13], [14]. Studies on the hot brines of the Red Sea were much more limited, and include the isolation and description of *Flexistipes sinusarabici* (phylum *Deferribacteres*) from the Atlantis II brine pool [15]. A recent 16S rDNA survey suggested the presence of similar microbial communities in Atlantis II and Discovery Deep brines [16], while a comparative metagenomic analysis revealed profound differences in the dominant bacterial groups in these environments [17]. The latter study also identified genes associated with degradation of aromatic compounds in Atlantis II [17].

**Figure 1:**
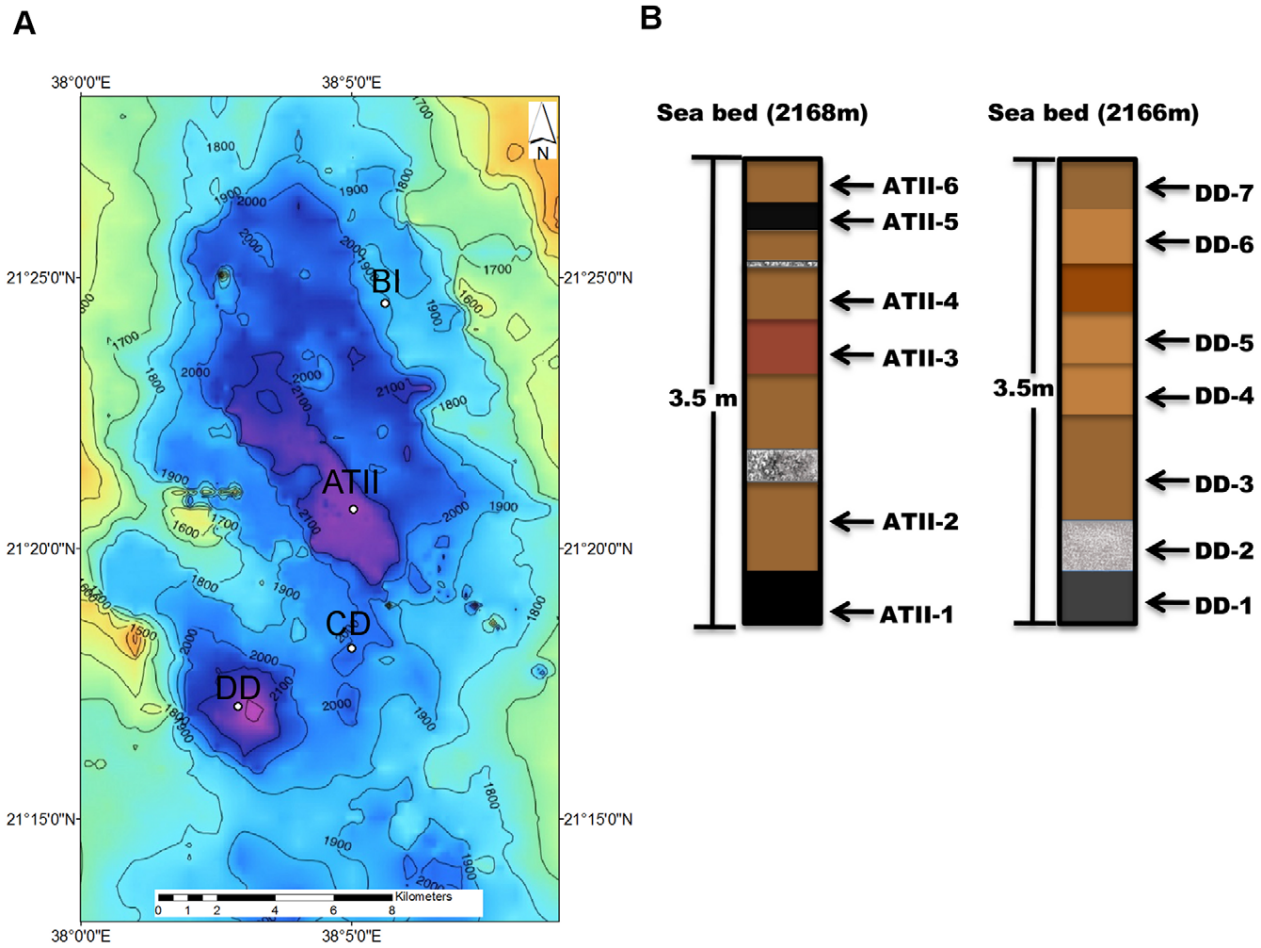
Sediment samples. (**1a**) ***Map of the sampling and water depth in the Atlantis II Deep area.*** The Atlantis II Deep area (between latitudes 21° 13′N and 21° 30′N and longitudes 37° 58′E and 38° 9′E) is in the central rift zone of the Red Sea, between Saudi Arabia and Sudan. The four sample sites are represented. (**1b**)**:** ***Schematic representation of the Atlantis II (ATII), Discovery (DD), Chain Deep (CD) and Brine-Influenced (BI) sediment cores.*** The sediment cores are represented individually. Each subsection in each core is presented to scale, and sections are numbered from bottom to top. See Table 1 for sampling and sample details. ATII (Atlantis II; with a total of six distinctive sediment sections), DD (Discovery Deep; with seven sections), CD (Chain Deep; one section), and adjacent brine sections (BI; two sections).

Whereas these previous studies have yielded insights into the composition and metabolic capabilities of microbial organisms in brine pools, the microbial assemblages that inhabit the sediments underlying the hot brine-pools in the Red Sea and it's relationship to sediment geochemistry have not been reported previously. Here we analyze and compare the prokaryotic microbiomes in several chemically and geologically distinct sediment sections of Atlantis II (ATII), Discovery (DD), Chain Deep (CD) and an adjacent brine-influenced (BI) site (Fig. 1) using 16S rDNA pyrotag sequencing. In this poorly explored marine environment, distinctively unique prokaryotic communities were observed in a sulfur-rich Atlantis II and a nitrogen-rich core section of Discovery Deep brine pool, providing insight into how geochemical components allocates ‘polyextremophiles’.

**Table 1:** Samples and sampling locations.

	Coordinates	Core length	Coring	Distinct	Water depth	Brine temp
		(m)		intervals	(m)	(°C)
Atlantis II (ATII)	21° 20.732′ N, 38° 05.039′ E	∼3.5	GGC	6 (ATII-1-ATII-6)	2168	68
Discovery Deep (DD)	21° 17.085′ N, 38° 02.904′ E	∼3.5	GGC	7 (DD1-DD7)	2180	45
Chain Deep (CD)	21° 18.155′ N, 38° 05.004′ E	∼4	GGC	1	1937	32
Brine Influenced (BI)	21° 24.532′ N, 38° 05.623′ E	0.37	MC	2 (BI-a, BI-b)	1856	22

## Results

### Chemical profiling of the sediments from brine pools

Sediment were collected from Atlantis II (ATII), Discovery (DD), Chain Deep (CD) and an adjacent brine-influenced (BI) site (Fig. 1 and Table 1) as described in materials and methods. Carbon Hydrogen Nitrogen & Sulfur (CHN&S) profiling showed that the analyzed brine sediments and the adjacent BI sediments differed greatly in C and S content, whereas little variation was found in H (1.4–1.9% w/w) or N (<0.10 to 0.12% w/w) content (Table 2). Sulfur content was highest in the ATII sediment sections (mean 8.3% w/w) with the highest value found in the deepest analyzed section (ATII-1; 19% w/w). The sulfur content was on average much lower in the other brine (0.6% in DD and <0.10% in CD) and adjacent (BI; 0.3–1.0% w/w) sediments. SO_4_^2−^ concentrations varied more between ATII sediments than % sulfur, but were also found to be highest in the deepest ATII-1 section (20% w/w). Far lower SO_4_^2−^ concentrations were measured in the other brine and BI sediments (Table 2). Carbon content on the other hand was highest in DD (∼7% w/w) and BI (8.3–8.7% w/w) followed by one order of magnitude lower values in ATII and CD sediments (∼0.8% w/w). The highest level of carbon was found in DD-2. Interestingly nitrogen (0.22% w/w) was only detected in the deepest section of DD (DD-1). CHN&S results are tabulated in Table 2.

**Table 2:** Chemical profiling of the sediments.

	ATII-1	ATII-2	ATII-3	ATII-4	ATII-5	ATII-6	DD-1	DD-2	DD-3	DD-4	DD-5	DD-6	DD-7	CD	BI-1	BI-2
%C	0.6	0.5	0.6	0.6	2.0	1.0	4.0	9.7	7.5	4.6	8.3	8.2	7.0	0.8	8.7	8.3
%H	0.6	1.6	1.8	1.6	1.8	1.8	1.0	1.6	1.5	1.6	1.5	1.2	1.4	1.9	1.5	1.4
%N	<0.10	<0.10	<0.10	<0.10	<0.10	<0.10	**0.22**	<0.10	<0.10	<0.10	<0.10	<0.10	<0.10	<0.10	<0.10	<0.10
%S	**19.1**	6.9	3.4	4.2	10.4	4.7	<0.1	0.6	0.4	1.3	1.4	0.4	0.3	<0.1	0.3	1
% SO_4_^2−^	**20.1**	1.8	0.2	0.1	**9**	1	1.3	0.04	0.01	0.04	0.1	0.05	0.08	0.03	0.08	0.05

### Pyrotag 16S rDNA data sets

A total of 1,012,178 reads were generated from the 16 brine and adjacent sediment samples and the overlying ATII water column (Table 3), ranging from a total of 14,006 for ATII-1 to 53,665 for ATII-6 (Table 3). The sediment reads were compared to 388,659 reads from the water column. Of the total sediment pyrotag library, 242,366 pyrotags represented bacteria and 377,425 represented archaea. The number of bacterial reads varied between 12,125 in ATII-1 and 22,540 in ATII-6. The number of archaeal reads generated varied between 1,624 in ATII-1 and 37,451 in DD-5. Unassigned reads (unknown) represented a relatively small proportion of the total pyrotags.

**Table 3:** Pyrotag 16S rDNA data sets.

	ATII-1	ATII-2	ATII-3	ATII-4	ATII-5	ATII-6	DD-1	DD-2	DD-3	DD-4	DD-5	DD-6	DD-7	CD	BI-1	BI-2	Total	
	highest S						highest N										(Sediment)	(water)
**Total**	14006	40452	28890	30079	45150	53665	36619	39029	38142	45080	42813	38918	40954	42131	43626	43965	623519	388659
**Archeal**	1624	28012	15636	13967	30232	30813	21652	25331	23003	28878	24067	24562	25010	27517	28404	28717	377425	91747
**Bacterial**	12125	12223	13078	15765	14737	22540	14787	13500	14931	15933	18449	14143	15714	14429	14990	15022	242366	295277
**Unknown**	257	217	176	347	181	312	180	198	208	269	297	213	230	185	232	226	3728	1635
DNA (ng)	259	290	310	300	285	225	115	300	200	453	600	150	250	520	153	250		

### Taxonomic assignment of OTUs to major archaeal groups

**Figure 2:**
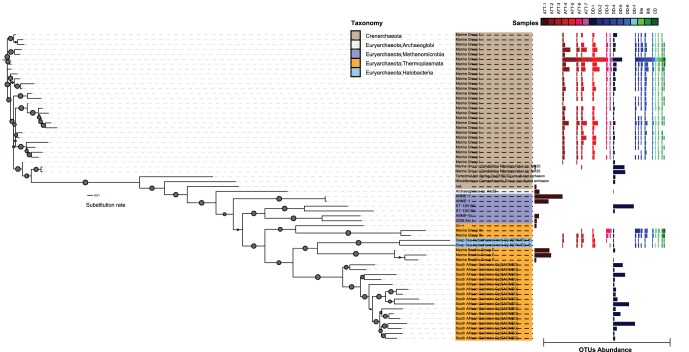
Taxonomic assignment and abundance of archaeal communities in ATII, DD, CD and BI sediment samples. Maximum-likelihood phylogenetic tree showing the taxonomic diversity and relative abundance of archaeal OTUs in all sediment samples. Bootstrap support values greater than 50% are indicated by the size of the circle on each branch. The taxonomically assigned OTUs are represented in ST-1.

The overall archaeal community structure differed greatly between the S-rich ATII-1, N-rich DD-1, and the remaining sediments (Fig. 2, Table 4). Out of 39 archaeal OTUs analyzed, 10 were detected in both water column and sediments (Table S1), 6 were detected only in the water column (data not shown) and 23 were only detected in the sediments (Table S1, Table 4). To exclude potential contamination from the overlying water column we focus on the sediment only taxa, that doesn’t exclude the fact that taxa shared in sediment and water may also be present in sediments. In the S-rich ATII-1, euryarchaeota clearly dominated the archaeal community (96%) with 50% of the archaeal tags in this section being assigned to Marine Benthic Group E (MBG-E; *Thermoplasmata*), note that MBG-E was not detected in the water column. Other abundant euryarchaeota in ATII-1 were *Methanomicrobia* belonging to ANME-1b (25%) and the *Methanosarcinales* clade GOM ArcI. Pyrotags of the latter groups were not detected in any of the other sediment sections or the water column (Fig. 2, Table 4).

**Table 4:** Taxonomic assignment of OTUs to major archaeal and bacterial groups.

Taxonomy-archaea	ATII-1	ATII-2	ATII-3	ATII-4	ATII-5	ATII-6	DD-1	DD-2	DD-3	DD-4	DD-5	DD-6	DD-7	CD	BI-1	BI-2	Water Col.^1^	Water Col.^2^
**I- Euryarchaeota**																		
IA- Thermoplasmata																		
1- Marine Benthic Group E	**50.4**	0.0	0.0	0.0	0.0	0.0	**0.2**	0.0	0.0	0.0	0.0	0.0	0.0	0.0	0.0	0.0	0.0	0.0
2- South African Goldmine Group	**1.5**	0.0	0.0	0.0	0.0	0.0	**14.8**	0.0	0.0	0.0	0.0	0.0	0.0	0.0	0.0	0.0	0.8	1.8
3- Thermoplasmatales Marine Group III	**0.0**	**0.9**	**0.9**	**0.1**	14.8	12.1	**1.0**	13.5	6.4	13.2	12.2	11.9	7.5	7.7	11.5	12.7	12.7	30.0
IB- Methanomicrobia																		
1- ANME-1 ANME-1b	**25.3**	0.0	0.0	0.0	0.0	0.0	0.0	0.0	0.0	0.0	0.0	0.0	0.0	0.0	0.0	0.0	0.0	0.0
2- Methanosarcinales GOM Arc I	**10.4**	0.0	0.0	0.0	0.0	0.0	0.0	0.0	0.0	0.0	0.0	0.0	0.0	0.0	0.0	0.0	0.0	0.0
3-Methanomicrobiales-Family (NA)	0	0.07	0.12	0.07	0.18	0.21	0.019	0.24	0.3	0.28	0.24	0.29	0.25	0.24	0.24	0.23	0.0	0.0
4-Methanomicrobiales-Methanospirillum	0	0.34	0.17	0.09	0.3	0.3	0.06	0.44	0.43	0.66	0.45	0.46	0.5	0.48	0.43	0.4	0.0	0.0
5- ST-12K10A	**0.1**	0.0	0.0	0.0	0.0	0.0	**41.9**	0.0	0.0	0.0	0.0	0.0	0.0	0.0	0.0	0.0	0.0	0.0
1C-Halobacteria																		
1-Halobacteriaceae-Halobacterium	0.0	0.0	0.0	0.0	0.0	0.0	**0.17**	0.0	0.0	0.0	0.0	0.0	0.0	0.0	0.0	0.0	0.0	0.0
2-Halobacteriaceae-Halococcus	0.0	0.0	0.0	0.0	0.0	0.0	**0.06**	0.0	0.0	0.0	0.0	0.0	0.0	0.0	0.0	0.0	0.0	0.0
3-Halobacteriaceae-Halomicrobium	0.0	0.0	0.0	0.0	0.0	0.0	**0.19**	0.0	0.0	0.0	0.0	0.0	0.0	0.0	0.0	0.0	0.0	0.0
4-Halobacteriaceae-Halorhabdus	0.0	0.0	0.0	0.0	0.0	0.0	**0.05**	0.0	0.0	0.0	0.0	0.0	0.0	0.0	0.0	0.0	0.0	0.0
5-Halobacteriaceae-Halosimplex	0.0	0.0	0.0	0.0	0.0	0.0	**0.1**	0.0	0.0	0.0	0.0	0.0	0.0	0.0	0.0	0.0	0.0	0.0
6-Halobacteriaceae-Genus (NA)	0.0	0.1	0.0	0.0	0.6	0.5	**2.7**	0.5	0.4	0.6	0.7	0.7	0.4	0.7	0.6	0.5	1.1	3.2
7-Halobacteriaceae-Natronomonas	0.0	0.0	0.0	0.0	0.0	0.0	**0.26**	0.0	0.0	0.0	0.0	0.0	0.0	0.0	0.0	0.0	0.0	0.0
8-MSP-41	4.0	0.0	0.0	0.0	0.0	0.0	**0.05**	0.0	0.0	0.0	0.0	0.0	0.0	0.0	0.0	0.0	0.0	0.0
1D-Archaeoglobi																		
**II- Crenarchaeota**	1.9	93.6	95.4	96.1	75.2	78.5	34.3	75.1	85.7	73.2	74.7	74.8	83.1	83.6	76.9	76.9	40.6	90.0
IIA-Marine Group I	0.0	0.0	0.0	0.08	0.02	0.05	**0.44**	0.01	0.03	0.01	0.04	0.02	0	0.04	0.02	0.01	0.0	0.0
IIB-Terrestrial Hot Spring	**50.4**	0.0	0.0	0.0	0.0	0.0	**0.2**	0.0	0.0	0.0	0.0	0.0	0.0	0.0	0.0	0.0	0.0	0.0
**Taxonomy-Bacteria**																		
Actinobacteria	**0.36**	10.07	**0.1**	9.04	10.65	13.32	**2.43**	12.79	13.01	12.67	12.62	12.89	13.24	13.1	13.1	13	3.08	7.55
Chloroflexi	**32.23**	1.3	2.86	1.46	1.06	1.22	2.19	1.45	1.3	1.37	1.63	1.25	1.25	1.5	1.33	1.15	4.97	12.48
Cyanobacteria	**0.43**	1.62	0.2	1.15	7	9.08	**22.4**	7.58	7.72	8.13	8.7	6.94	8.14	8.5	7.35	8.34	4.33	16.44
Deferribacteres	10.36	6.34	11.41	5.98	3.15	4.36	6.34	4.43	4.15	4.08	4.85	4.72	4.35	4.5	4.78	4.37	11.4	17.04
∝-proteobacteria	**1.15**	54.03	42.27	53.27	34.83	39.17	25.75	39.35	40.54	38.56	38.41	39.16	40.05	36.6	38.4	38.97	34.7	39.9
δ-proteobacteria	6.46	7.89	15.55	8.02	3.57	3.92	3.78	4.31	4.39	4.22	3.93	4.24	4.08	4.24	4.25	4.02	12.36	19.95
γ-proteobacteria	**4**	13.34	17	13.56	17.52	22.1	21.77	23.07	22.1	23.74	22.72	23.93	22.35	24.3	23.8	23.4	22.24	30
Fusobacteria	**20**	0	0	0	0	0	**0.83**	0	0	0	0	0	0	0	0	0	0	0
OP1	**15**	0	0	0	0	0	**5.11**	0	0	0	0	0	0	0	0	0	0	0

Water Col.^1^: Average OTU in the water column

Water Col.^2^: Highest OTU in the water column

Similarly, euryarchaeota predominated the archaeal community in the N-rich DD-1 section (65%). Here, an unclassified family of the ST-12K10A lineage (SA1 group) represented the most abundant archaeal group (42%), whereas pyrotags of this archaeal family were very rare (0.1% in ATII-1) or absent in all other analyzed sediment sections and the water column. Uncultured *Thermoplasmata* of the South African Goldmine (SAG) Group, were found to be ten times more abundant in DD-1 (15%) than in the S-rich ATII-1, whereas MBG-E were 250 times less abundant than in the ATII-1 sections. Marine Group III (MG-III) *Thermoplasmatales*, which were not recovered from ATII-1, represented 0.2% of the total archaeal pyrotags in DD-1 (Table 4). Although not highly abundant, we detect a diverse and exclusive *Halobacteriales*-related pyrotags only in the N-rich Discovery Deep brine-pool sediments (Fig. 2, Table 4).

Crenarchaeota assigned to Marine Group I (MGI) comprised only 1.9% in the S-rich ATII-1 and 34.3% in the N-rich DD-1 sediments, whereas members of this group represented the most abundant archaea in all other sediment sections (81.6±8.2%) including the water column (Table 4). *Methanomicrobiales*-*Methanospirillum*, *Halobacteriales* Deep Sea Hydrothermal Vent Group 6 and Terrestrial Hot Spring *Crenarchaeota* were detected in the remaining sediments but not in the water column (Table 4).

**Figure 3:**
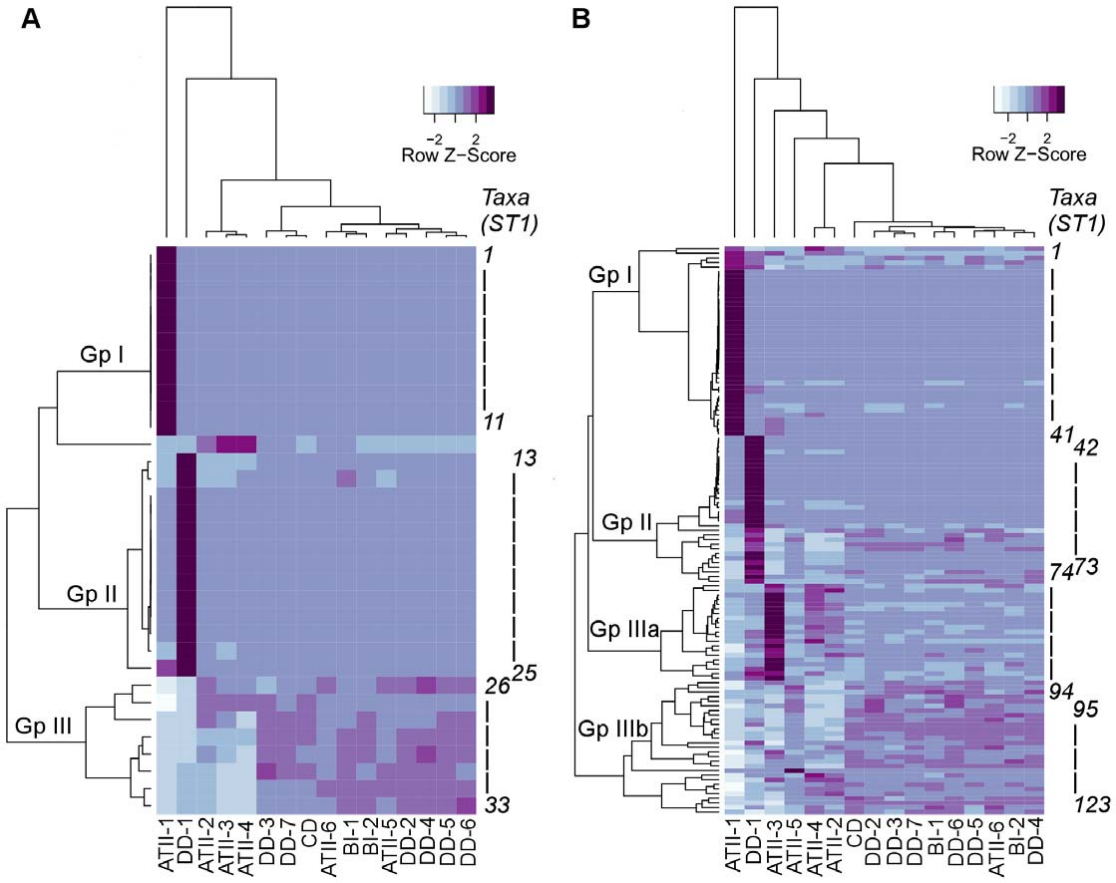
Heat map representation of the relative abundance of major archaeal (3a) and bacterial (3b) groups in ATII, DD, CD and BI sediment samples. Heat map showing a comparative taxonomic assignment of OTUs to major archaeal taxa (at the genus level). The different taxa represented in each group are illustrated in Table S1. The dendrogram show the hierarchical clustering of the different sediment sections based on the relative abundance of the OTUs in each section. The Z-scores shows the rescaling of the value of the row based on its mean and standard deviation. Group I (ATII-1), group II (DD-1), group IIIa (ATII-2-5), and group IIIb (all other samples) are presented. The taxonomically assigned OTUs are represented in ST-1.

Relative abundance-based hierarchical clustering of the 16S rDNA pyrotags assigned to major taxonomic groups allowed us to categorize the sediment archaeal communities into three major and distinct groups (Fig. 3A, Table S1) and to detect a set of sediment/brine specific archaeal OTUs (23) that were not detected in the overlying water column (Table S1). Group I (12 OTUs, of which 10 are sediment specific) was unique to the S-rich Atlantis II sample (ATII-1), group II (13 OTUs, of which 10 are sediment specific) was characteristic for the N-rich Discovery sample (DD-1), and group III (8 OTUs, of which 4 are sediment specific) reflected the composition of the remaining sediment samples.

### Taxonomic assignment of OTUs to major bacterial groups

Similar to archaea, the bacterial community structure greatly differed between the S-rich ATII-1, N-rich DD-1, compared to the remaining overlying and adjacent sediments. Out of 171 bacterial OTUs, 75 were detected in both water column and sediments (Table S1), 42 were detected only in the water column (data not shown) and 48 were only detected in the sediments (Table S1, Table 4).

In the S-rich ATII-1, *Chloroflexi* accounted for 32% of the assigned bacterial OTUs, with the order *Anaerolineales* predominating (Table 4, Fig. S1). The distribution of *Chloroflexi* was relatively constant, but at a much lower abundance (1.71±0.98%) in all other analyzed sediment sections. *Fusobacteria* (20%) and the OP1 division (15%) were two of the major bacterial phyla in the S-rich ATII-1 sample. The latter group also comprised 5% of the bacterial OTUs in DD-1 but was insignificant elsewhere (including the water column). In contrast to their high abundance in all the other samples, *Alpha*- and *Gammaproteobacteria* constituted only 1% and 4% of bacterial OTUs in ATII-1, respectively.

In the N-rich DD-1, *Alphaproteobacteria* dominated the bacterial community constituting 25% of bacterial OTUs, followed by *Cyanobacteria* with 22%. In other DD sediments, the latter phylum was far less prominent, and this variability is largely due to the contributions of an unassigned family related to *Prochlorococcus* (21%). *Prochlorococcus* constituted 4% (±8) of water column OTUs, and therefore the overlying water column could have compromised this sample.

Based on the relative abundance of 16S rDNA pyrotags and their assignment to major bacterial groups a set of sediment/brine specific bacterial OTUs (48) were identified (Table S1). Additionally, three distinct sediment groups were observed in our samples. Group I (41 OTUs, of which 27 are sediment specific) are enriched in the S-rich Atlantis II sample (ATII-1), group II (31 OTUs, of which 15 are sediment specific) corresponds to the N-rich Discovery sample (DD-1), group IIIa (20 OTUs, of which 3 are sediment specific) in samples ATII-2 to ATII-5, while group IIIb (34 OTUs, of which 3 are sediment specific) reflects the combination of taxa found in all other samples (Fig. 3B, Table S1). Rare bacterial communities were determined by selecting OTUs that accounted for less than 1% of reads in all the samples. Comparative analysis of the less abundant bacterial genera leads to the same basic division into four consortia (Fig. S2, Table S2). The distinctive bacterial communities in the S-rich Atlantis II and N-rich Discovery samples are discussed in relation to sulfur and methane cycling below.

### Comparative analysis of microbial community in the Red Sea sediments with diverse oceanic systems

**Figure 4:**
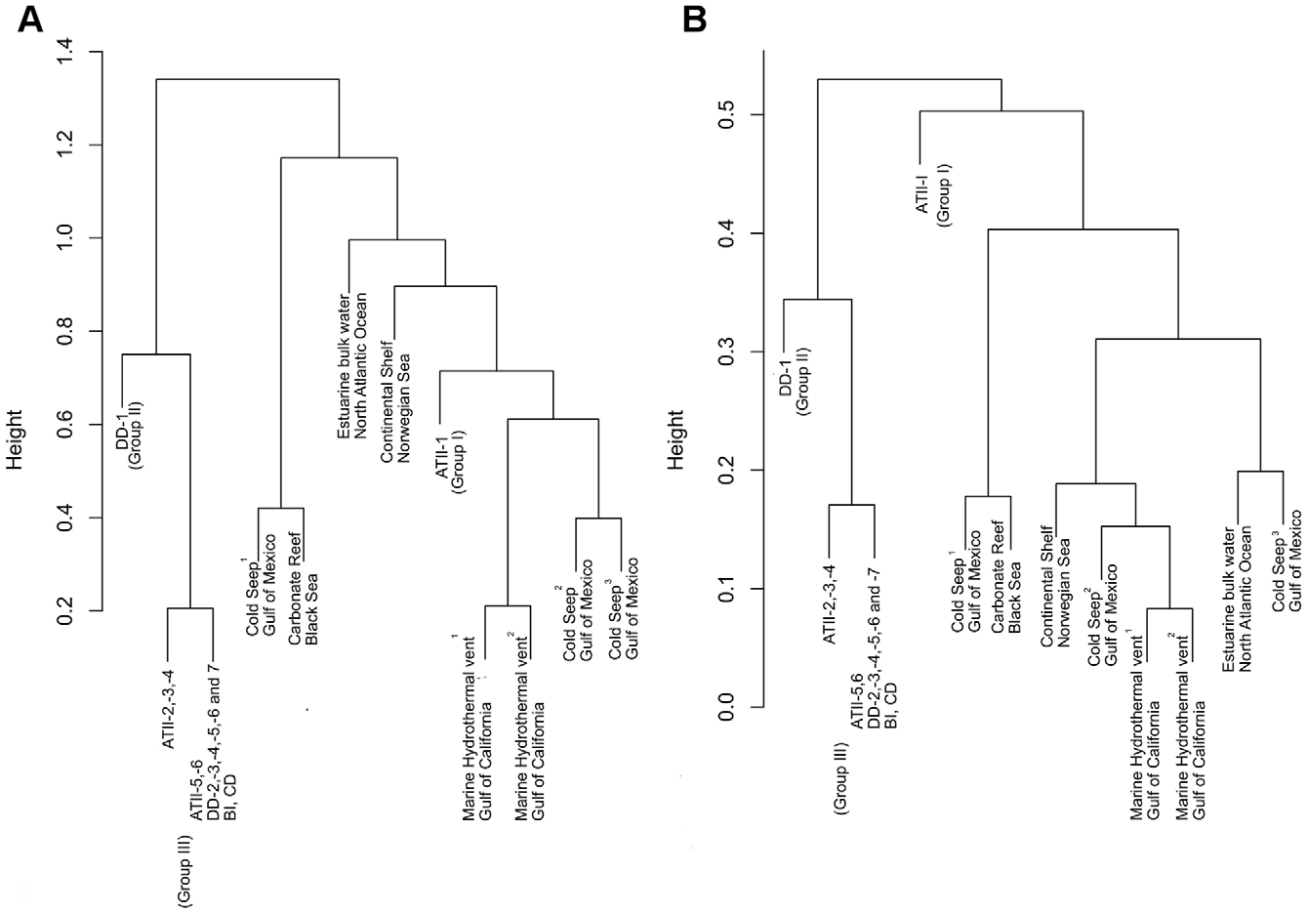
Cluster dendrogram illustrating the relationship of the (4a) archaeal and (4b) bacterial communities in the Red Sea to those in other oceanic systems. The dendrogram shows the complete linkage hierarchical clustering of the different sediment sections based on the relative abundance of the OTUs in each section. The height indicates the relative distances between the datasets. The data of other oceanic systems from the Guaymas Methane Seep (ICM GMS) project included *Estuarine bulk water, North Atlantic Ocean* (ICM GMS 1–2): oxic sediment from the White Oak River; *Cold Seep^1^ Gulf of Mexico* (ICM GMS 3–4): microbial mat; *Cold Seep^2,3^ Gulf of Mexico* (ICM GMS 5–8): sediments from anoxic deep-sea hydrocarbon seeps in the Gulf of Mexico; *Continental shelf, Norwegian Sea* (ICM GMS 13–14): an oxic microbial mat from the continental shelf at the Storegga seep enrichment in the Norwegian Sea; *Carbonate reef Black Sea* (ICM GMS 15–16): an anoxic microbial mat from the Black Sea; *Marine hydrothermal vent^1–2^, Gulf of California* (ICM GMS 17–20): microbial mat from the Gulf of California, oxic hydrothermal vent from Guaymas Basin.

To assess the uniqueness and diversity of the microbial communities present in the Atlantis II brine-system sediments, we compared the pyrotags recovered in this study with a global survey of sub-seafloor microbial communities. The 16S rDNA tag sequences that were used for this comparison are publicly available through the VAMPS website http://vamps.mbl.edu (project title ICM_GMS). We compared our data with oxic sediments from the White Oak River estuary in the North Atlantic Ocean [18], anoxic deep-sea hydrocarbon seeps from both a microbial mat and sediments [19] in the Gulf of Mexico, a microbial mat from the oxic Storegga seep enrichment on the continental shelf in the Norwegian Sea, an anoxic microbial mat from a carbonate reef in the Black Sea, and a microbial mat from an oxic hydrothermal vent in the Guaymas Basin in the Gulf of California. These oceanic sediments were surveyed as part of the ‘Microbial Census of Methane and Hydrocarbon Seep Sediments in Relation to Geochemical and Temperature Gradients’. Heat map analysis revealed four sample groups based on the unique microbial consortia present: I) ATII-1, II) DD-1, IIIa) ATII-2 to ATII-5 and IIIb) ATII-6, DD-2 to DD-7 and CD and BI (Fig. 3). A clustering analysis was used to compare and contrast our data with 16S rRNA (V6) obtained from the other oceanic systems. With respect to both Archaea and Bacteria, groups II and III clustered in one clade (Fig. 4A and 4B). Both the bacterial and archaeal assemblages at ATII-1 showed greater similarity with the consortia in the methane and hydrocarbon seep systems. Notably, ATII-1 showed a closer resemblance to the communities at marine hydrothermal vents in the Gulf of California (Fig. 4A).

## Discussion

### Unique microbial taxa in the brine pool sediments that are enriched in the S-rich Atlantis II and the N-rich Discovery brine-pool sediments

We identify the unique microbial communities in sediments from the three Red Sea brine pool sites. To exclude potential contamination from the overlying water column, we focus on the 48 sediment/brine specific bacterial taxa and 23 archaeal taxa. Although, the microbial communities in sediments from three Red Sea brine pool deeps and a brine-influenced site displayed very similar distributions of abundant and rare microbial groups, the deepest S-rich sediment section from Atlantis II and an N-rich section from Discovery Deep each revealed unique archaeal and bacterial communities. Clustering of 16S rDNA pyrotags from each sediment sample into major bacterial and archaeal groups on the basis of relative abundance and taxonomic assignment (Fig. 3, Table S1 and Fig. S2), allowed us to discern three major assemblages, comprising (I) taxa that are unique for S-rich ATII-1, (II) taxa unique for N-rich DD-1, (IIIa) taxa that are restricted to samples ATII-2 to ATII-4, and (IIIb) taxa that are found in all the remaining samples (i.e., ATII-5 and ATII-6, CD, BI and DD-2 to DD-7 (Fig. 3A). Note, that the water column is clustered into a fourth group, with a closer resemblance to group III (data not shown). Such clustering suggests vertical and horizontal variability in the prokaryotic community structure within the Atlantis II deep-sea brine system. Comparison with data from other oceanic sites revealed that the prokaryotic community in the ATII-1 is more closely related to prokaryotes found in methane and hydrocarbon seep sediments (Fig. 4). The detection of OTUs from known typical marine prokaryotic groups (e.g., MGI, *Cyanobacteria* and SAR11) might result from accumulation in the sediments of non-active or dead cells or even cellular debris previously active in the water column. It may also be a result of contamination from the overlying water column. However, we attempted to exclude such potential contaminants by subtracting ATII water column taxa from sediment taxa, and we have stressed on the sediment/brine-specific taxa in this study. However, this does not exclude that these OTUs may belong to sediment-clades or possibly new Atlantis II brine system clades. For example, members of the MGI seem to dominate the archaeal community in most sediment layers. Members of this group have been previously found within the brine-systems, but seemed to be restricted to the brine-seawater interface [12], probably reflecting the standard communities found in seawater above the brine. MGI is dominant in deep-sea water and surface (oxic) sediments, although populations in these two biotopes should not be assumed as being identical [20], [21] There are specific sediment phylotypes, distinct from seawater relatives [20].

Although we have only utilized one sediment core at each sample point, our results can be generalized to the entire brine because the physical conditions within the spatial dimensions of the brine pool are uniform. Performing a single DNA extraction protocol may also be a limitation in our study, suggesting a representation of, rather than the entire, microbiota in each site.

The composition of the prokaryotic community in the S-rich section of Atlantis II sediments suggests metabolism based on sulfate reduction and methane consumption. On the other hand, the diversity and exclusive detection of selected *Halobacteriales* in the N-rich Discovery Deep sediment may be related to its characteristic elemental profile (discussed below).

### The S-rich subsection of the Atlantis II brine-pool sediment sample harbors a unique consortium of prokaryotes putatively involved in sulfate-reduction coupled with anaerobic oxidation of methane

The oxidation of methane in anoxic marine sediments is mediated by consortia of methane-oxidizing and sulfate-reducing archaea and bacteria [22], in a process described in previous studies [23]. The exclusive combined presence of pyrotag related to archaeal and bacterial sulfate-reducing genera (*Archaeoglobus* [24], and *Desulfovermiculus* sp. (*Deltaproteobacteria*)) and anaerobic methane-oxidizing archaeal clusters ANME-1 (*Methanosarcinales* and *Methanomicrobiales*) [25], ANME-2 and ANME-3 (*Methanosarcinales*), strongly argues for an exclusive inhabitance of microbial sulfate reduction coupled with anaerobic oxidation of methane in the S-rich ATII section (Fig. 3B).

The distribution of *Methanomicrobia* in the S-rich Atlantis II sample is also unique, and quite distinct from those in the remaining samples. *Methanosarcinales* species were exclusively identified in the S-rich ATII-1. *Methanomicrobia* were identified in all the sediments analyzed, but their abundance and detailed composition varied widely. *Methanomicrobia* were most abundant in the S-rich ATII-1 and N-rich DD-1 samples, but accounted for <1% of reads in the remaining samples. The ANME-1b clade and the *Methanosarcinales* clade GOM-Arc-1 constituted the majority of *Methanomicrobia* in the S-rich ATII-1, while ST-12K10A (SA1) dominated the N-rich DD-1 sample, which was previously identified in Shaban Deep and brine-sea water interface of Discovery Deep [14]. Interestingly, in these two samples, hydrogenotrophic *Methanomicrobiales* (DD-1) and acetoclastic *Methanosarcinales* (ATII-1) were mutually exclusive. Presence of sulfate reducers would explain these differences as they would likely be partially involved in anaerobic methane oxidizing consortia with ANME and would simultaneously outcompete and exclude all methanogenic groups with the exception of *Methanosarcinales* [25], [26]. This implies distinctive styles of methane production in the S-rich Atlantis II and N-rich Discovery sediment sections.

Overall, our comparative taxonomic assignments of OTUs that defined the four distinct microbial assemblages (Fig. 3B) reveal a unique consortium of methanogenic, ANME, and sulfate-reducing microbes that are distinct in the environments of S-rich ATII-1, N-rich DD-1 and the remaining samples.

### Diversity of denitrifying microbes in the N-rich subsection of the Discovery Deep brine-pool sediment

Nitrification requires several groups of microorganisms and play a significant role in the nitrogen cycle [27].

Group II archaea were predominantly detected in DD-1 (Table 4 and Figs. 2B, 3B) and included potential ammonium oxidizers such as *Crenarchaeum* of the terrestrial hot spring group (Table 4) [28]–[30]. On the other hand, archaea known to be capable of reducing oxidized nitrogen species were also exclusively identified in DD-1, such as *Halobacteriaceae; Natronomonas* sp., *Halococcus* sp., *Halobacterium* sp., *Halorabdus* sp., *Halosimplex* sp., and an unassigned MSP41 genus (Fig. 2). A previous study documented an abundance of *Halobacteriaceae* in the Atlantis II and Discovery Deep overlying water column [16]. However, our analysis did not identify such abundance of *Halobacteriaceae*. These are capable of reducing nitrate to nitrite during anaerobic respiration [28]–[30]. Note that, in contrast to other analyzed brine and non-brine sediments, the archaea in Group II, which are present in DD-1, all cluster among ammonium oxidizers. Interestingly, the S-rich Atlantis II sections (ATII-1), was the only sample that had no detectable *Halobacteriaceae* related to nitrogen cycling. The diversity and detection of archaea previously known to play a role in nitrogen cycling in the N-rich Discovery sample implies an active role specifically in nitrogen regulation in this sediment section.

## Conclusion

This study identifies three major microbial consortiums in sediments of brine and brine-influenced sites in the Red Sea. Based on taxonomic assignment of 16S rDNA OTUs to major bacterial and archaeal groups, the characteristic distributions of both the abundant and the rare taxa was revealed. A sulfur-rich Atlantis II and a nitrogen-rich Discovery Deep section harbored distinct prokaryotic assemblages that differed furthermore from those found in all other sediment samples examined. The exclusive combined presence of sulfate-reducing and anaerobic methane-oxidizing prokaryotes strongly argues for an exclusive importance of microbial sulfate reduction coupled with anaerobic oxidation of methane in the S-rich ATII section. Additionally, the diversity of denitrifying microorganisms in the N-rich Discovery section implies an active role in nitrogen cycling. More in depth characterization of the physical and chemical properties of these sediment sections combined with further metagenomics and metatranscriptomes could shed further insights into microbial metabolic processes and the cycling of (in)organic matter in these under-sampled extreme environments.

## Materials and Methods

### Sample collection

The sediment samples from the Atlantis II, Discovery Deep, and Chain Deep brines, as well as from the brine-influenced site used for this study were collected during the 2^nd^ KAUST/WHOI Red Sea Expedition of the *RV Aegaeo* in April 2010 (see Table 1 for details about the samples and sampling locations). All necessary permits were obtained for the described field studies. Between 3.5 and 4 m-long gravity cores (Benthos Instruments, KC Denmark) were obtained from the brine sediments, whereas the short BI core was obtained by multicoring. The water depth of the brine settings varied only between 1937 and 2168 m, but the brines differed greatly in temperature, being highest at ATII (68°C), followed by DD (45°C) and CD (32°C) (Table 1). The BI site was slightly shallower (1856 m water depth) and the temperature (22°C) was lowest of all settings. Water samples were collected from the overlying ATII water, at different depth 50 m, 200 m, 700 m and 1500 m (Ferreira *et. al*., submitted). Water depth in the Atlantis II Deep area was obtained during the cruise from a bathymetric survey with single-beam echo sounder on R/V *Oceanus* (Fig. 1A).

Cores were divided into sections (numbered from bottom to top) and different sediment sections were studied separately (Fig. 1B). In ATII, six distinct sections of the sediment core were macroscopically identified (Fig. 1B). The bottom 50 cm of the ATII core (ATII-1) consisted of black slurry. ATII-2 was dominated by brown, ATII-3 by reddish-brown, and ATII-4 by brown clay. ATII-5 was composed of black slurry, while brownish slurry sediments (ATII-6) were found at the top of the core facing the lower convective layer of the pool. The seven DD sediment samples were less macroscopically distinct than the ATII sections (Fig. 1B). For the brine-influenced sediments, BI-1 comprised the top half (0–5 cm) and BI-2 bottom 5 cm of the core, although the two samples were not macroscopically distinct. All sediment samples were immediately frozen upon recovery, and kept at −20°C until further analysis.

### Chemical analysis

Profiling of the elements C, H, N and S was performed by oxidation of the samples by flash combustion using a Thermo FlashEA 1112 elemental analyzer. The combusting gases were then separated by passage through a chromatographic column using He as a carrier gas, and were detected with thermal conductivity detector. Sulfate level was measured by ion chromatography.

### DNA extraction, pyrosequencing of 16S rDNA amplicons and computational analysis

Genomic DNA was extracted 0.5–1 gm of sediment with the PowerSoil® DNA Isolation Kit (MO-BIO, Calsbad, CA), the total amount of DNA isolated from the sediments is presented in Table 2. DNA was used for PCR amplification of environmental 16S rRNA genes (16S rDNA) for archaeal and bacterial taxonomic assignment after Sogin *et. al*. (2006) [31]. The primers utilized in this study were V6-V4 primer pairs (bacterial: 1046R CGACRRCCATGCANCACCT and 518F CCAGCAGCYGCGGTAAN; archaeal: 1048R CGrCrGCCATGyACCwC, arc517F1 GCCTAAAGCATCCGTAGC, arc517F2 GCCTAAArCGTyCGTAGC, arc517F3 GTCTAAAGGGTCyGTAGC, arc517F4 GCTTAAAGnGTyCGTAGC, and arc517F5 GTCTAAArCGyyCGTAGC. The amplicons recovered were then subjected to pyrosequencing by 454 GS FLX Titanium technology (454 Life Sciences).

Phylogenetic analysis of the sequence tags and subsequent taxonomic assignments were performed using the resources on the Visualization and Analysis of Microbial Population Structures (VAMPS) website, hosted by the Josephine Bay Paul Center, MBL, Woods Hole (http://vamps.mbl.edu/resources/databases.php).

To infer phylogenetic relationships among the identified Operational Taxonomic Units (OTUs), an initial taxonomic classification into bacterial or archaeal groups was performed. The OTUs were estimated by clustering the 910,068 raw 16S reads with an identity threshold >0.97% using USEARCH (http://www.drive5.com/usearch/). The seed (longest sequence that is at least 0.97% similar to all other sequences in the cluster), was utilized as the representative sequence (i.e., the OTU) of that cluster, resulting into a non-redundant set of 837 bacterial OTUs and 63 archaeal OTUs. For each domain, all OTUs across the different samples were aligned using MUSCLE [32]. The two generated multiple sequence alignments were visually inspected and manually tuned. A maximum-likelihood phylogenetic tree was then estimated based on the refined alignment under the GTR model [33] using FastTree [34]. Finally, the estimated tree was taxonomically annotated, and the normalized abundance levels of each OTU in the different samples were graphically represented on the trees as bars stemming from the corresponding OTU using iToL [35].

Heat maps were generated using data statistically filtered by a two-tailed Fisher's exact test (FET). The normalized read value for each taxon in a sample was compared to those of other samples, and to the totals of remaining taxa. Statistical analyses were performed using R (http://cran.r-prroject.org) [36]. Results were filtered for a p-value threshold <0.05. The frequency-normalized values of the filtered statistically significant data were used to create heat maps using the ‘enhanced heat map function’ from the R package gplots (http://cran.r-project.org/package=gplots). Complete linkage hierarchical clustering was used across the samples. The Pearson correlation distance matrix was calculated between the taxa. We also performed Euclidean distance Ward clustering approach and Spearman's correlation, which did not alter the clustering of the data (data not shown).

## Supporting Information

Figure S1 **Taxonomic assignment and relative abundance of bacterial OTUs in ATII, DD, CD and BI sediment samples.** Maximum-likelihood phylogenetic tree showing the bacterial taxonomic diversity in sediments and the abundance for each OTU in all sediment samples.
(EPS)

Figure S2 **Heat map and dendrogram of low-abundance bacterial groups based on recovery of 16S rDNA tags.** Heat map generated up to the genera level, represented by less than 1% of bacterial reads. The taxonomically assigned OTUs are represented in Table S1.
(TIF)

Table S1 **The archaeal assigned OTUs (from** **Fig. 3A** **) and the bacterial assigned OTUs (from** **Fig. 3B** **) are tabulated.** OTUs detected only in sediments are in bold.
(DOCX)

Table S2 **The rare bacterial assigned OTUs (from SF2) are tabulated.**
(DOCX)
