# Supplementary figures and images for: Unique Prokaryotic Consortia in Geochemically Distinct Sediments from Red Sea Atlantis II and Discovery Deep Brine Pools
Source: PLoS One. 2012 Aug 20;7(8):e42872. doi: 10.1371/journal.pone.0042872 (PMC3423430; doi:10.1371/journal.pone.0042872)

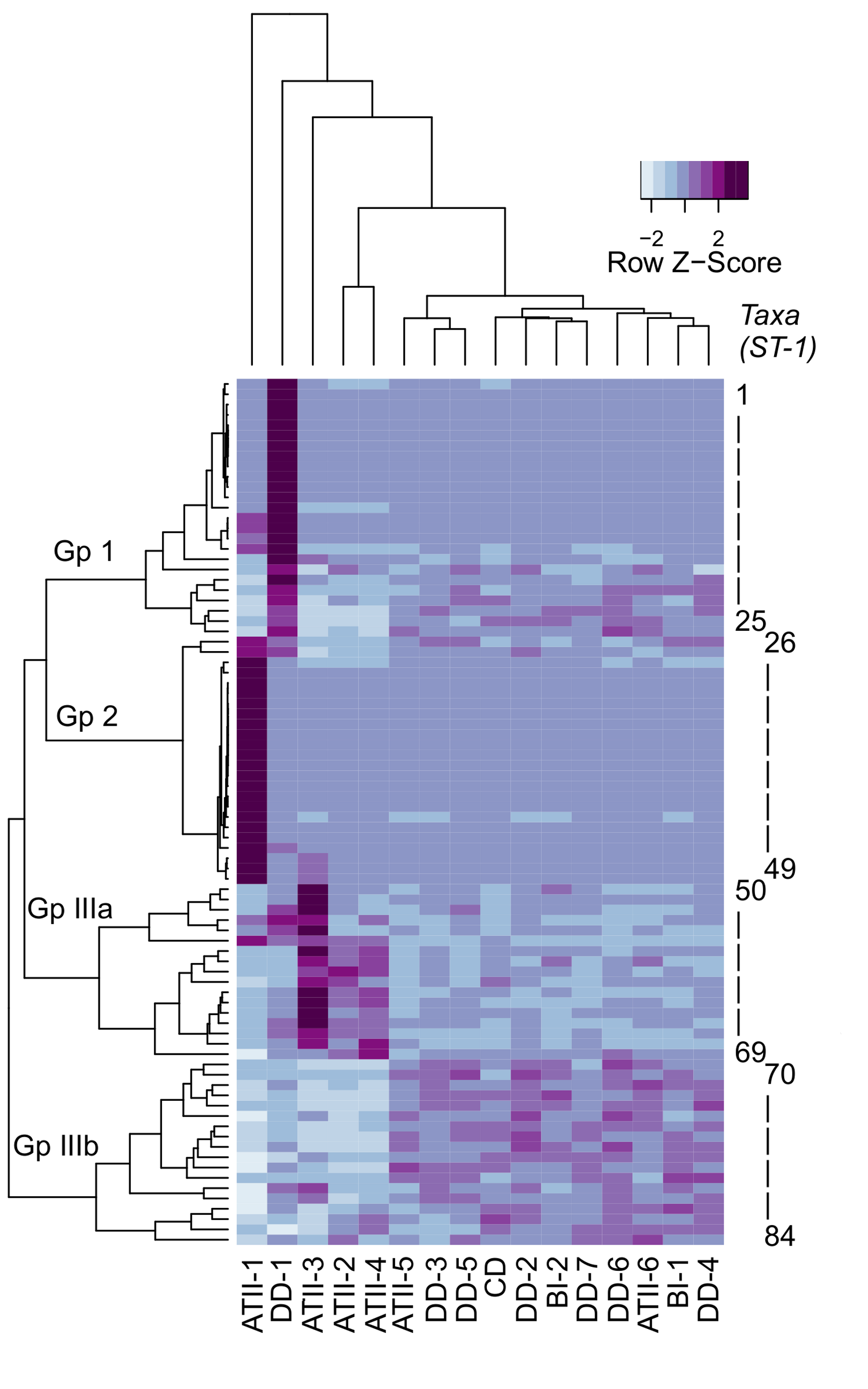

Supplement: Figure S2 — Heat map and dendrogram of low-abundance bacterial groups based on recovery of 16S rDNA tags. Heat map generated up to the genera level, represented by less than 1% of bacterial reads. The taxonomically assigned OTUs are represented in Table S1. (TIF) [file pone.0042872.s002.tif]
