# Supplementary material for: Unique Prokaryotic Consortia in Geochemically Distinct Sediments from Red Sea Atlantis II and Discovery Deep Brine Pools
Source: PLoS One. 2012 Aug 20;7(8):e42872. doi: 10.1371/journal.pone.0042872 (PMC3423430; doi:10.1371/journal.pone.0042872)
Supplement: Table S1 — The archaeal assigned OTUs (from Fig. 3A ) and the bacterial assigned OTUs (from Fig. 3B ) are tabulated. OTUs detected only in sediments are in bold. (DOCX) [file pone.0042872.s003.docx]

**Supplemental Table 1:** The archaeal assigned OTUs (from Fig. 3a) and the bacterial assigned OTUs (from Fig. 3b) are tabulated. OTUs detected only in sediments are in bold.

|  | Archaeal Assigned OTUs (Figure 3a) |
| --- | --- |
|  | Group I (ATII-1) |
| **1** | **Euryarchaeota;Thermoplasmata;Thermoplasmatales;20c−4;genus_NA** |
| **2** | **Euryarchaeota;Archaeoglobi;Archaeoglobales;Archaeoglobaceae;genus_NA** |
| **3** | **Euryarchaeota;Archaeoglobi;Archaeoglobales;Archaeoglobaceae;Archaeoglobus** |
| **4** | **Euryarchaeota;Thermoplasmata;Marine_Benthic_Group_E;family_NA;genus_NA** |
| **5** | **Euryarchaeota;Methanomicrobia;Methanosarcinales;Methanosaetaceae;Methanosaeta** |
| **6** | **Euryarchaeota;Methanomicrobia;ANME−1;family_NA;genus_NA** |
| **7** | **Euryarchaeota;Methanomicrobia;ANME−1;ANME−1b;genus_NA** |
| **8** | **Euryarchaeota;Methanomicrobia;Methanosarcinales;ANME−2a−2b;genus_NA** |
| **9** | **Euryarchaeota;Methanomicrobia;Methanosarcinales;GOM_Arc_I;genus_NA** |
| **10** | **Euryarchaeota;Methanomicrobia;Methanosarcinales;Methanosarcinaceae;ANME−3** |
| 11 | Unknown;phylum_NA;class_NA;orderx_NA;family_NA;genus_NA |
| 12 | Crenarchaeota;AK59;orderx_NA;family_NA;genus_NA |
|  | Group II (DD-1) |
| 13 | Euryarchaeota;Halobacteria;Halobacteriales;Halobacteriaceae;genus_NA |
| **14** | **Euryarchaeota;Halobacteria;Halobacteriales;Deep_Sea_Euryarcheotic_Group;genus_NA** |
| **15** | **Euryarchaeota;Thermoplasmata;Thermoplasmatales;KTK_4A;genus_NA** |
| **16** | **Euryarchaeota;Halobacteria;Halobacteriales;MSP41;genus_NA** |
| **17** | **Euryarchaeota;Halobacteria;Halobacteriales;Halobacteriaceae;Halococcus** |
| **18** | **Euryarchaeota;Halobacteria;Halobacteriales;Halobacteriaceae;Halobacterium** |
| **19** | **Euryarchaeota;Halobacteria;Halobacteriales;Halobacteriaceae;Halomicrobium** |
| **20** | **Euryarchaeota;Halobacteria;Halobacteriales;Halobacteriaceae;Halorhabdus** |
| **21** | **Euryarchaeota;Halobacteria;Halobacteriales;Halobacteriaceae;Halosimplex** |
| **22** | **Euryarchaeota;Methanomicrobia;ST−12K10A;family_NA;genus_NA** |
| 23 | Euryarchaeota;Thermoplasmata;South_African_Goldmine_Group;family_NA;genus_NA |
| **24** | **Crenarchaeota;Terrestrial_Hot_Spring_Group;orderx_NA;family_NA;genus_NA** |
| 25 | Crenarchaeota;Miscellaneous_Crenarchaeotic_Group;orderx_NA;family_NA;genus_NA |
|  | Group III (ATII-2 to ATII-6, DD-2 to DD-7, CD and BI) |
| **26** | **Euryarchaeota;Halobacteria;Halobacteriales;Deep_Sea_Hydrothermal_Vent_Group_6;genus_NA** |
| 27 | Crenarchaeota;Marine_Group_I;orderx_NA;family_NA;genus_NA |
| 28 | Crenarchaeota;Marine_Benthic_Group_A;orderx_NA;family_NA;genus_NA |
| **29** | **Euryarchaeota;Methanomicrobia;Methanomicrobiales;family_NA;genus_NA** |
| **30** | **Euryarchaeota;Methanomicrobia;Methanomicrobiales;Methanospirillaceae;Methanospirillum** |
| 31 | Crenarchaeota;pSL12;orderx_NA;family_NA;genus_NA |
| 32 | Euryarchaeota;Thermoplasmata;Thermoplasmatales;Marine_Group_III;genus_NA |
| 33 | Euryarchaeota;Thermoplasmata;Thermoplasmatales;Marine_Group_II;genus_NA |
|  |  |
|  | Bacterial assigned OTUs (Figure 3b) |
|  | Group I (ATII-1) |
| 1 | Unknown;phylum_NA;class_NA;orderx_NA;family_NA;genus_NA |
| 2 | Proteobacteria;Betaproteobacteria;Burkholderiales;Burkholderiaceae;Ralstonia |
| 3 | Firmicutes;Clostridia;Halanaerobiales;family_NA;genus_NA |
| **4** | **Cyanobacteria;Cyanobacteria;SubsectionI;family_NA;genus_NA** |
| 5 | Proteobacteria;Deltaproteobacteria;Bdellovibrionales;Bacteriovoraceae;genus_NA |
| **6** | **Spirochaetes;Spirochaetes;Spirochaetales;Spirochaetaceae;genus_NA** |
| **7** | **Fusobacteria;Fusobacteria;orderx_NA;family_NA;genus_NA** |
| **8** | **Proteobacteria;Deltaproteobacteria;Desulfobacterales;Desulfobacteraceae;genus_NA** |
| **9** | **Proteobacteria;Gammaproteobacteria;Thiotrichales;Thiotrichaceae;Leucothrix** |
| **10** | **Chloroflexi;Anaerolineae;Anaerolineales;Anaerolinaceae;genus_NA** |
| 11 | Chloroflexi;class_NA;orderx_NA;family_NA;genus_NA |
| **12** | **Proteobacteria;Deltaproteobacteria;Syntrophobacterales;Syntrophobacteraceae;genus_NA** |
| **13** | **Nitrospirae;Nitrospira;Nitrospirales;Nitrospiraceae;genus_NA** |
| **14** | **Deferribacteres;Deferribacteres;Deferribacterales;Unassigned;Caldithrix** |
| **15** | **Deferribacteres;Deferribacteres;Deferribacterales;Deferribacteraceae;Deferribacter** |
| **16** | **Firmicutes;Clostridia;Clostridiales;Syntrophomonadaceae;Dethiobacter** |
| **17** | **Firmicutes;Clostridia;orderx_NA;family_NA;genus_NA** |
| **18** | **Nitrospirae;Nitrospira;Nitrospirales;Nitrospiraceae;Thermodesulfovibrio** |
| **19** | **OP8;class_NA;orderx_NA;family_NA;genus_NA** |
| **20** | **Proteobacteria;Alphaproteobacteria;Rhizobiales;Aurantimonadaceae;Aurantimonas** |
| **21** | **Proteobacteria;Gammaproteobacteria;Aeromonadales;Aeromonadaceae;Aeromonas** |
| **22** | **Proteobacteria;Gammaproteobacteria;Chromatiales;Chromatiaceae;Alishewanella** |
| 23 | Chloroflexi;Anaerolineae;Anaerolineales;family_NA;genus_NA |
| **24** | **Proteobacteria;Deltaproteobacteria;Desulfarculales;Desulfarculaceae;genus_NA** |
| 25 | OP3;class_NA;orderx_NA;family_NA;genus_NA |
| **26** | **Actinobacteria;Actinobacteria;Actinomycetales;Propionibacteriaceae;Propionibacterium** |
| **27** | **Bacteroidetes;Sphingobacteria;Sphingobacteriales;Rhodothermaceae;genus_NA** |
| 28 | Proteobacteria;Alphaproteobacteria;Caulobacterales;Caulobacteraceae;Caulobacter |
| **29** | **Proteobacteria;Gammaproteobacteria;Alteromonadales;Shewanellaceae;Shewanella** |
| **30** | **Firmicutes;Bacilli;Bacillales;Staphylococcaceae;Staphylococcus** |
| **31** | **Proteobacteria;Gammaproteobacteria;Thiotrichales;Piscirickettsiaceae;Mariprofundus** |
| **32** | **OP1;class_NA;orderx_NA;family_NA;genus_NA** |
| **33** | **Firmicutes;Bacilli;Lactobacillales;Streptococcaceae;Streptococcus** |
| 34 | Chloroflexi;Dehalococcoidetes;Unassigned;Unassigned;Dehalogenimonas |
| 35 | Proteobacteria;Deltaproteobacteria;Desulfobacterales;family_NA;genus_NA |
| 36 | phylum_NA;class_NA;orderx_NA;family_NA;genus_NA |
| **37** | **Proteobacteria;Betaproteobacteria;Burkholderiales;Burkholderiaceae;Cupriavidus** |
| 38 | Proteobacteria;Gammaproteobacteria;Enterobacteriales;Enterobacteriaceae;genus_NA |
| 39 | Proteobacteria;Alphaproteobacteria;Rhizobiales;Bradyrhizobiaceae;Bradyrhizobium |
| **40** | **Proteobacteria;Gammaproteobacteria;Thiotrichales;family_NA;genus_NA** |
| 41 | Proteobacteria;Deltaproteobacteria;orderx_NA;family_NA;genus_NA |
|  | Group II (DD-1) |
| **42** | **Proteobacteria;Alphaproteobacteria;Rhodospirillales;Rhodospirillaceae;Thalassospira** |
| 43 | Bacteroidetes;Flavobacteria;Flavobacteriales;family_NA;genus_NA |
| 44 | Proteobacteria;Alphaproteobacteria;Sphingomonadales;Sphingomonadaceae;Sphingomonas |
| 45 | Proteobacteria;Gammaproteobacteria;Pseudomonadales;Pseudomonadaceae;Pseudomonas |
| **46** | **Proteobacteria;Alphaproteobacteria;Rhizobiales;Methylobacteriaceae;Methylobacterium** |
| **47** | **Bacteroidetes;Flavobacteria;Flavobacteriales;Flavobacteriaceae;Formosa** |
| **48** | **Proteobacteria;Betaproteobacteria;Burkholderiales;Burkholderiaceae;Limnobacter** |
| **49** | **Planctomycetes;Unassigned;Unassigned;Unassigned;Scalindua** |
| 50 | Proteobacteria;Gammaproteobacteria;Alteromonadales;Pseudoalteromonadaceae;Pseudoalteromonas |
| **51** | **Bacteroidetes;Flavobacteria;Flavobacteriales;Cryomorphaceae;genus_NA** |
| 52 | Verrucomicrobia;Opitutae;Puniceicoccales;Puniceicoccaceae;Coraliomargarita |
| 53 | Proteobacteria;Gammaproteobacteria;Alteromonadales;Alteromonadaceae;SAR92 |
| **54** | **Proteobacteria;Alphaproteobacteria;Sphingomonadales;Sphingomonadaceae;Sphingobium** |
| **55** | **Proteobacteria;Alphaproteobacteria;Sphingomonadales;Erythrobacteraceae;Erythrobacter** |
| 56 | Cyanobacteria;Cyanobacteria;SubsectionI;Unassigned;Synechococcus |
| **57** | **Bacteroidetes;Flavobacteria;Flavobacteriales;Cryomorphaceae;Owenweeksia** |
| **58** | **Proteobacteria;Deltaproteobacteria;Desulfovibrionales;Desulfohalobiaceae;Desulfovermiculus** |
| **59** | **Nitrospirae;Nitrospira;Nitrospirales;Nitrospiraceae;Leptospirillum** |
| **60** | **Proteobacteria;Gammaproteobacteria;Oceanospirillales;Alcanivoracaceae;Alcanivorax** |
| 61 | Proteobacteria;Alphaproteobacteria;Rhodospirillales;Rhodospirillaceae;Defluviicoccus |
| 62 | Proteobacteria;Betaproteobacteria;Burkholderiales;Alcaligenaceae;genus_NA |
| **63** | **Proteobacteria;Deltaproteobacteria;Bdellovibrionales;Bdellovibrionaceae;genus_NA** |
| 64 | Actinobacteria;Actinobacteria;Actinomycetales;family_NA;genus_NA |
| 65 | Proteobacteria;Gammaproteobacteria;Oceanospirillales;family_NA;genus_NA |
| 66 | Proteobacteria;Gammaproteobacteria;Oceanospirillales;Halomonadaceae;Halomonas |
| **67** | **Proteobacteria;Gammaproteobacteria;Pseudomonadales;Moraxellaceae;Acinetobacter** |
| **68** | **Proteobacteria;Deltaproteobacteria;Desulfuromonadales;family_NA;genus_NA** |
| 69 | Cyanobacteria;Cyanobacteria;SubsectionI;Unassigned;Prochlorococcus |
| 70 | Proteobacteria;Alphaproteobacteria;Rickettsiales;SAR116;genus_NA |
| 71 | Bacteroidetes;Sphingobacteria;Sphingobacteriales;Flammeovirgaceae;Marinoscillum |
| 72 | Proteobacteria;Gammaproteobacteria;Alteromonadales;Idiomarinaceae;Idiomarina |
| 73 | Proteobacteria;Gammaproteobacteria;Alteromonadales;Alteromonadaceae;Marinobacter |
|  | Group IIIa |
| 74 | Proteobacteria;Gammaproteobacteria;Thiotrichales;Piscirickettsiaceae;Piscirickettsia |
| 75 | Deferribacteres;Deferribacteres;Deferribacterales;family_NA;genus_NA |
| **76** | **OP11;class_NA;orderx_NA;family_NA;genus_NA** |
| 77 | Nitrospirae;Nitrospira;Nitrospirales;Nitrospiraceae;Nitrospira |
| 78 | Acidobacteria;Acidobacteria_Gp26;Unassigned;Unassigned;Gp26 |
| 79 | TM6;class_NA;orderx_NA;family_NA;genus_NA |
| 80 | Acidobacteria;Acidobacteria;Acidobacteriales;Acidobacteriaceae;genus_NA |
| 81 | Proteobacteria;Deltaproteobacteria;SAR324;family_NA;genus_NA |
| 82 | Gemmatimonadetes;Gemmatimonadetes;orderx_NA;family_NA;genus_NA |
| 83 | Proteobacteria;Gammaproteobacteria;Xanthomonadales;Sinobacteraceae;genus_NA |
| 84 | Proteobacteria;Deltaproteobacteria;Desulfobacterales;Nitrospinaceae;Nitrospina |
| 85 | Proteobacteria;Alphaproteobacteria;Rickettsiales;family_NA;genus_NA |
| **86** | **Chlamydiae;Chlamydiae;Chlamydiales;Chlamydiaceae;genus_NA** |
| 87 | Deferribacteres;Deferribacteres;Deferribacterales;SAR406;genus_NA |
| 88 | Chloroflexi;SAR202;orderx_NA;family_NA;genus_NA |
| 89 | Proteobacteria;Deltaproteobacteria;Myxococcales;family_NA;genus_NA |
| **90** | **Chlamydiae;Chlamydiae;Chlamydiales;Simkaniaceae;genus_NA** |
| 91 | Chlamydiae;Chlamydiae;Chlamydiales;Simkaniaceae;Rhabdochlamydia |
| 92 | Proteobacteria;Gammaproteobacteria;Thiotrichales;Piscirickettsiaceae;genus_NA |
| 93 | OD1;class_NA;orderx_NA;family_NA;genus_NA |
| 94 | Chloroflexi;Anaerolineae;Anaerolineales;Anaerolinaceae;Bellilinea |
|  | Group IIIb |
| 95 | Verrucomicrobia;class_NA;orderx_NA;family_NA;genus_NA |
| 96 | Planctomycetes;Phycisphaerae;Phycisphaerales;Phycisphaeraceae;genus_NA |
| 97 | Proteobacteria;Gammaproteobacteria;Oceanospirillales;Oceanospirillaceae;genus_NA |
| **98** | **Planctomycetes;Planctomycetacia;Planctomycetales;Planctomycetaceae;Rhodopirellula** |
| 99 | Planctomycetes;Planctomycetacia;Planctomycetales;Planctomycetaceae;Planctomyces |
| 100 | Verrucomicrobia;Opitutae;Puniceicoccales;Puniceicoccaceae;Puniceicoccus |
| 101 | Planctomycetes;Planctomycetacia;Planctomycetales;Planctomycetaceae;genus_NA |
| **102** | **Planctomycetes;Phycisphaerae;Phycisphaerales;Phycisphaeraceae;Phycisphaera** |
| 103 | Spirochaetes;Spirochaetes;orderx_NA;family_NA;genus_NA |
| **104** | **Bacteroidetes;Flavobacteria;Flavobacteriales;Flavobacteriaceae;genus_NA** |
| 105 | Proteobacteria;Gammaproteobacteria;Oceanospirillales;Oceanospirillaceae;Oceanobacter |
| 106 | Proteobacteria;Alphaproteobacteria;orderx_NA;family_NA;genus_NA |
| 107 | Proteobacteria;Gammaproteobacteria;Alteromonadales;Alteromonadaceae;Haliea |
| 108 | Proteobacteria;Alphaproteobacteria;Rhodobacterales;Rhodobacteraceae;genus_NA |
| 109 | Actinobacteria;Actinobacteria;Acidimicrobiales;Iamiaceae;Iamia |
| 110 | Proteobacteria;Gammaproteobacteria;orderx_NA;family_NA;genus_NA |
| 111 | Proteobacteria;Gammaproteobacteria;Salinisphaerales;Salinisphaeraceae;genus_NA |
| 112 | Proteobacteria;Gammaproteobacteria;Oceanospirillales;Oceanospirillaceae;Pseudospirillum |
| 113 | Proteobacteria;Gammaproteobacteria;Alteromonadales;Alteromonadaceae;Alteromonas |
| 114 | Firmicutes;Bacilli;Bacillales;Bacillaceae;Bacillus |
| 115 | Proteobacteria;Gammaproteobacteria;Legionellales;Coxiellaceae;Coxiella |
| 116 | Proteobacteria;Alphaproteobacteria;Rickettsiales;SAR11;Pelagibacter |
| 117 | Proteobacteria;Gammaproteobacteria;Oceanospirillales;SAR86;genus_NA |
| 118 | Proteobacteria;Alphaproteobacteria;Rhodospirillales;Rhodospirillaceae;genus_NA |
| 119 | Proteobacteria;Alphaproteobacteria;Rickettsiales;SAR11;genus_NA |
| 120 | Proteobacteria;Gammaproteobacteria;Thiotrichales;Thiotrichaceae;Thiothrix |
| 121 | Actinobacteria;Actinobacteria;Acidimicrobiales;family_NA;genus_NA |
| 122 | Verrucomicrobia;Opitutae;Puniceicoccales;Puniceicoccaceae;genus_NA |
| 123 | Cyanobacteria;class_NA;orderx_NA;family_NA;genus_NA |
