# Supplementary material for: Unique Prokaryotic Consortia in Geochemically Distinct Sediments from Red Sea Atlantis II and Discovery Deep Brine Pools
Source: PLoS One. 2012 Aug 20;7(8):e42872. doi: 10.1371/journal.pone.0042872 (PMC3423430; doi:10.1371/journal.pone.0042872)
Supplement: Table S2 — The rare bacterial assigned OTUs (from SF2) are tabulated. (DOCX) [file pone.0042872.s004.docx]

**Supplemental Table 2:** The rare bacterial assigned OTUs (from SF2) are tabulated.

|  | Rare Bacterial Assigned OTUs (SF-2) |
| --- | --- |
|  | Group II (DD-1) |
| 1 | Bacteroidetes;Flavobacteria;Flavobacteriales;Cryomorphaceae;Owenweeksia |
| 2 | Proteobacteria;Alphaproteobacteria;Sphingomonadales;Sphingomonadaceae;Sphingobium |
| 3 | Verrucomicrobia;Opitutae;Puniceicoccales;Puniceicoccaceae;Coraliomargarita |
| 4 | Bacteroidetes;Flavobacteria;Flavobacteriales;Cryomorphaceae;genus_NA |
| 5 | Proteobacteria;Betaproteobacteria;Burkholderiales;Burkholderiaceae;Limnobacter |
| 6 | Proteobacteria;Alphaproteobacteria;Rhizobiales;Methylobacteriaceae;Methylobacterium |
| 7 | Bacteroidetes;Flavobacteria;Flavobacteriales;Flavobacteriaceae;Formosa |
| 8 | Planctomycetes;Unassigned;Unassigned;Unassigned;Scalindua |
| 9 | Proteobacteria;Gammaproteobacteria;Alteromonadales;Alteromonadaceae;SAR92 |
| 10 | Proteobacteria;Alphaproteobacteria;Sphingomonadales;Sphingomonadaceae;Sphingomonas |
| 11 | Bacteroidetes;Flavobacteria;Flavobacteriales;family_NA;genus_NA |
| 12 | Proteobacteria;Alphaproteobacteria;Sphingomonadales;Erythrobacteraceae;Erythrobacter |
| 13 | Proteobacteria;Deltaproteobacteria;Desulfuromonadales;family_NA;genus_NA |
| 14 | Proteobacteria;Deltaproteobacteria;Desulfovibrionales;Desulfohalobiaceae;Desulfovermiculus |
| 15 | Nitrospirae;Nitrospira;Nitrospirales;Nitrospiraceae;Leptospirillum |
| 16 | Proteobacteria;Gammaproteobacteria;Oceanospirillales;Alcanivoracaceae;Alcanivorax |
| 17 | Cyanobacteria;Cyanobacteria;SubsectionI;family_NA;genus_NA |
| 18 | Proteobacteria;Alphaproteobacteria;Rhodospirillales;Rhodospirillaceae;Defluviicoccus |
| 19 | Proteobacteria;Betaproteobacteria;Burkholderiales;Alcaligenaceae;genus_NA |
| 20 | Proteobacteria;Gammaproteobacteria;Alteromonadales;Idiomarinaceae;Idiomarina |
| 21 | Proteobacteria;Gammaproteobacteria;Alteromonadales;Alteromonadaceae;Marinobacter |
| 22 | Bacteroidetes;Sphingobacteria;Sphingobacteriales;Flammeovirgaceae;Marinoscillum |
| 23 | Proteobacteria;Gammaproteobacteria;Oceanospirillales;Halomonadaceae;Halomonas |
| 24 | Proteobacteria;Deltaproteobacteria;Bdellovibrionales;Bdellovibrionaceae;genus_NA |
| 25 | Actinobacteria;Actinobacteria;Actinomycetales;family_NA;genus_NA |
|  | Group I (ATII-1) |
| 26 | Firmicutes;Clostridia;Halanaerobiales;family_NA;genus_NA |
| 27 | Proteobacteria;Deltaproteobacteria;Bdellovibrionales;Bacteriovoraceae;genus_NA |
| 28 | Firmicutes;Bacilli;Bacillales;Staphylococcaceae;Staphylococcus |
| 29 | Proteobacteria;Gammaproteobacteria;Alteromonadales;Shewanellaceae;Shewanella |
| 30 | Spirochaetes;Spirochaetes;Spirochaetales;Spirochaetaceae;genus_NA |
| 31 | Proteobacteria;Deltaproteobacteria;Desulfobacterales;Desulfobacteraceae;genus_NA |
| 32 | Firmicutes;Clostridia;orderx_NA;family_NA;genus_NA |
| 33 | Proteobacteria;Gammaproteobacteria;Chromatiales;Chromatiaceae;Alishewanella |
| 34 | Firmicutes;Clostridia;Clostridiales;Syntrophomonadaceae;Dethiobacter |
| 35 | OP8;class_NA;orderx_NA;family_NA;genus_NA |
| 36 | Nitrospirae;Nitrospira;Nitrospirales;Nitrospiraceae;genus_NA |
| 37 | Nitrospirae;Nitrospira;Nitrospirales;Nitrospiraceae; Thermodesulfovibrio |
| 38 | Proteobacteria;Gammaproteobacteria;Aeromonadales;Aeromonadaceae;Aeromonas |
| 39 | Actinobacteria;Actinobacteria;Actinomycetales;Prionibacteriaceae;Propionbacterium |
| 40 | Chloroflexi;class_NA;orderx_NA;family_NA;genus_NA |
| 41 | Firmicutes;Bacilli;Lactobacillales;Streptococcaceae;Streptococcus |
| 42 | Proteobacteria;Gammaproteobacteria;Thiotrichales;Thiotrichaceae;Leucothrix |
| 43 | Proteobacteria;Deltaproteobacteria;Desulfobacterales;family_NA;genus_NA |
| 44 | Proteobacteria;Alphaproteobacteria;Caulobacterales;Caulobacteraceae;Caulobacter |
| 45 | Proteobacteria;Betaproteobacteria;Burkholderiales;Burkholderiaceae;Cupriavidus |
| 46 | Proteobacteria;Gammaproteobacteria;Thiotrichales;Piscirickettsiaceae;Mariprofundus |
| 47 | Proteobacteria;Gammaproteobacteria;Enterobacteriales;Enterobacteriaceae;genus_NA |
| 48 | Proteobacteria;Alphaproteobacteria;Rhizobiales;Bradyrhizobiaceae;Bradyrhizobium |
| 49 | Proteobacteria;Gammaproteobacteria;Thiotrichales;family_NA;genus_NA |
|  | Group IIIa (ATII-2-ATII-5) |
| 50 | Chlamydiae;Chlamydiae;Chlamydiales;Simkaniaceae;Rhabdochlamydia |
| 51 | Chlamydiae;Chlamydiae;Chlamydiales;Simkaniaceae;genus_NA |
| 52 | Proteobacteria;Gammaproteobacteria;Thiotrichales;Piscirickettsiaceae;genus_NA |
| 53 | OD1;class_NA;orderx_NA;family_NA;genus_NA |
| 54 | Chloroflexi;Anaerolineae;Anaerolineales;Anaerolinaceae;Bellilinea |
| 55 | Proteobacteria;Betaproteobacteria;Burkholderiales;Burkholderiaceae;Ralstonia |
| 56 | Proteobacteria;Gammaproteobacteria;Xanthomonadales;Sinobacteraceae;genus_NA |
| 57 | Proteobacteria;Gammaproteobacteria;Thiotrichales;Piscirickettsiaceae;Piscirickettsia |
| 58 | Deferribacteres;Deferribacteres;Deferribacterales;family_NA;genus_NA |
| 59 | Proteobacteria;Deltaproteobacteria;Myxococcales;family_NA;genus_NA |
| 60 | Acidobacteria;Acidobacteria_Gp26;Unassigned;Unassigned;Gp26 |
| 61 | TM6;class_NA;orderx_NA;family_NA;genus_NA |
| 62 | Acidobacteria;Acidobacteria;Acidobacteriales;Acidobacteriaceae;genus_NA |
| 63 | Proteobacteria;Deltaproteobacteria;Desulfobacterales;Nitrospinaceae;Nitrospina |
| 64 | Verrucomicrobia;Verrucomicrobiae;Verrucomicrobiales;Verrucomicrobiaceae;genus_NA |
| 65 | Chlamydiae;Chlamydiae;Chlamydiales;Chlamydiaceae;genus_NA |
| 66 | Proteobacteria;Gammaproteobacteria;Legionellales;Coxiellaceae;Coxiella |
|  | Group IIIb (ATII-6, -7, DD-2 to DD-DD7, CD, BI) |
| 67 | Planctomycetes;Planctomycetacia;Planctomycetales;Planctomycetaceae;Rhodopirellula |
| 68 | Proteobacteria;Gammaproteobacteria;Oceanospirillales;Oleiphilaceae;Oleiphilus |
| 69 | Spirochaetes;Spirochaetes;orderx_NA;family_NA;genus_NA |
| 70 | Planctomycetes;Phycisphaerae;Phycisphaerales;Phycisphaeraceae;Phycisphaera |
| 71 | Proteobacteria;Deltaproteobacteria;Desulfobacterales;Nitrospinaceae;Nitrospinaceae |
| 72 | Verrucomicrobia;Opitutae;Puniceicoccales;Puniceicoccaceae;Puniceicoccus |
| 73 | Proteobacteria;Gammaproteobacteria;Alteromonadales;Alteromonadaceae;Haliea |
| 74 | Planctomycetes;Planctomycetacia;Planctomycetales;Planctomycetaceae;genus_NA |
| 75 | Planctomycetes;Planctomycetacia;Planctomycetales;Planctomycetaceae;Planctomyces |
| 76 | Proteobacteria;Gammaproteobacteria;Oceanospirillales;Oceanospirillaceae;Pseudospirillum |
| 77 | Planctomycetes;Phycisphaerae;Phycisphaerales;Phycisphaeraceae;genus_NA |
| 78 | Proteobacteria;Gammaproteobacteria;Oceanospirillales;Oceanospirillaceae;genus_NA |
| 79 | Proteobacteria;Gammaproteobacteria;Oceanospirillales;family_NA;genus_NA |
| 80 | Verrucomicrobia;class_NA;orderx_NA;family_NA;genus_NA |
| 81 | Verrucomicrobia;Opitutae;Puniceicoccales;Puniceicoccaceae;genus_NA |
| 82 | Proteobacteria;Gammaproteobacteria;Thiotrichales;Thiotrichaceae;Thiothrix |
| 83 | Cyanobacteria;class_NA;orderx_NA;family_NA;genus_NA |
| 84 | Actinobacteria;Actinobacteria;Acidimicrobiales;Iamiaceae;Iamia |
